# Immune composition of the mononuclear cell fraction of human umbilical cord blood

**DOI:** 10.3389/fimmu.2025.1614230

**Published:** 2025-08-01

**Authors:** Karen Kikuta, Esmond Lee, Talia Menezes, Hannah Fung, Alvaro Amorin, Aditi Agrawal, Theodore L. Roth, Matthew Porteus

**Affiliations:** ^1^ Center for Definitive and Curative Medicine, Stanford University School of Medicine, Stanford, CA, United States; ^2^ Institute for Stem Cell Biology and Regenerative Medicine, Stanford University School of Medicine, Stanford, CA, United States; ^3^ Bioprocessing Technology Institute (BTI), Agency for Science, Technology and Research (ASTAR), Singapore, Singapore; ^4^ Department of Biology, School of Humanities and Sciences, Stanford University, Stanford, CA, United States; ^5^ Chan Zuckerberg BioHub, San Francisco, CA, United States; ^6^ Department of Pathology, School of Medicine, Stanford, CA, United States; ^7^ Division of Pediatric Hematology, Oncology, and Stem Cell Transplantation and Regenerative Medicine, Stanford University School of Medicine, Stanford, CA, United States

**Keywords:** Peripheral Blood Mononuclear cells (PBMCs), umbilical cord blood (UCB), single-cell RNA sequencing (scRNA-seq), flow cytometry, immune maturation, neonatal immune system, cell therapy

## Abstract

Despite its therapeutic potential and unique immunological properties, the immune composition of umbilical cord blood lacks consistent and comprehensive characterizations. Human umbilical cord blood (UCB) is often discarded after delivery and is difficult to obtain for research purposes. Furthermore, most research on UCB is focused on properties of CD34+ hematopoietic stem cells for transplantation. The Binns Program for Cord Blood Research at Stanford University has the unique advantage of regular collection and isolation of mononuclear cells (MNC) from UCB donors. This study provides a robust characterization of the immune subset compositions of the CD34-negative MNC fraction of UCB (n=50). The study also compares the UCB data to adult peripheral blood (PB) mononuclear cells to identify differences in immune maturity. Using flow cytometry and single-cell RNA sequencing (scRNA-Seq), we analyzed UCB and adult PB MNC samples to characterize the cell surface protein and transcriptomic profiles of different immune subsets. Our study findings bring a higher-definition understanding of the unique immunological properties of umbilical cord blood. Study findings reveal a distinct immune profile in UCB, such as a higher average percentage of CD19 B Lymphocytes, CD4 T Cells, CD4 Naive T Cells, CD4 Recent Thymic Emigrants, CD8 Naive T Cells, CD8 Recent Thymic Emigrants, and CD19 Naive B Cells compared to adult PB. Additionally, there were fewer CD19 Memory B Cells in UCB compared to PB. The scRNA-Seq showed concordance in the proportion of immune cell types but captured more differentiated subtypes of cells. Additionally, scRNA-Seq showed unique clustering patterns in UCB, which reflect cell types that converge in adulthood as the immune system matures. These analyses yield the intriguing possibility that the immune heterogeneity of individuals at birth gives way to more stereotyped immune subsets as the immune system is exposed to the external environment and undergoes maturation. Overall, our findings provide a robust characterization of MNC UCB immune subsets and insights into how immune function develops from birth to adulthood.

## Introduction

Human Peripheral Blood Mononuclear cells (PBMCs) contain immune cells essential to innate and adaptive immunity (1). While conducting immune surveillance in systemic circulation, cells can also traffic to secondary lymphoid organs and tissues in response to infection and inflammation. Immune challenges over the lifetime of an individual modifies the immune system and leads to mature cells with immune memory (2). One way to understand a mature immune system is to understand its point of origin at birth. Mononuclear cells (MNCs) from Umbilical Cord Blood (UCB) provide the opportunity to study the naive immune system. A few studies have investigated the properties of UCB MNCs, typically focusing on the hematopoietic stem and progenitor cell (HSPC) population (3–8) and making comparisons with bone marrow (9–11) derived HSPCs, which have been the gold standard in transplantation.

From an immunological perspective, early studies investigating the phenotype of UCB MNCs identified populations such as T- and B-lymphocytes, as well as NK cells (5). They showed that lymphocytes appeared to be phenotypically immature (6, 12). Notably, these studies were published over 20 years ago before technological advances allowed high-dimensional analysis of component cell populations. In recent years, single-cell transcriptomics was used to analyze the expression patterns of known marker genes of nucleated cord blood cells (13). Despite recent publications and the continued recognition of the promise of UCB research (11), comprehensive characterizations of the MNC populations of UCB are still lacking.

The Binns Program for Cord Blood Research gave us the opportunity of obtaining a large number of human UCB on a weekly basis (14). Annually, the program collects hundreds of UCB samples, and its established MNC isolation protocol has led to the distribution of UCB products to over 20 laboratories and over 60 researchers around the Stanford University campus. We studied the immune subsets found in 50 UCB donors by flow cytometry and paired this with single-cell transcriptomics (scRNA-Seq) to gain deeper insight into the complexity of the MNC fraction. Additionally, we compared the immune subtype composition of UCB to adult peripheral blood (PB) MNCs to understand changes that take place in immune maturity.

## Materials and methods

### Study design

Immunophenotyping of mononuclear cells (MNCs) using flow cytometry was performed on 50 umbilical cord blood (UCB) and 22 adult peripheral blood (PB) samples. Additional analysis with 3 UCB and 3 PB samples was performed using RNA-sequencing for a more detailed characterization. Similar studies performed by D’Arena G et al. (6) and Mantri S. et al. (15), as well as resource availability (laboratory space, finances, and time), were used as a reference to determine sample size.

### Isolation of mononuclear cells

UCB MNCs were isolated from the UCB of term deliveries (≥34 weeks of gestation) at Lucile Packard Children’s Hospital-Stanford. UCB collections were performed through the Binns Program for Cord Blood Research with donor consent and institutional review board approval. Within 24 hours of collection, MNCs of UCB were obtained by density gradient separation of whole blood (Ficoll Paque Plus, GE Healthcare; 400g, room temperature, 30 minutes, deceleration off), followed by ammonium chloride red blood cell lysis (9:1 NH4Cl lysis buffer to cell suspension). Some UCB samples underwent additional processing to isolate CD34-negative MNCs by labeling with the human CD34 Microbead Kit Ultrapure according to manufacturer protocol (Miltenyi Biotec, San Diego, CA, USA) (14). Adult peripheral blood (PB) was collected from the Stanford Blood Center (SBC). Within 24 hours of collection, whole blood was diluted by 1:1 ratio with MACs buffer (1x PBS, EDTA, FBS). MNCs of PB were then obtained by density gradient separation (Ficoll Paque Plus, GE Healthcare; 400g, room temperature, 30 minutes, deceleration off), followed by ammonium chloride red blood cell lysis (9:1 NH4Cl lysis buffer to cell suspension). While our initial aims were to capture mononuclear cells, multinucleated cells such as Neutrophils found in the MNC fraction were also included as part of the study.

### Immunophenotyping of CD34-negative mononuclear cells with flow cytometry

**Figure 1:**
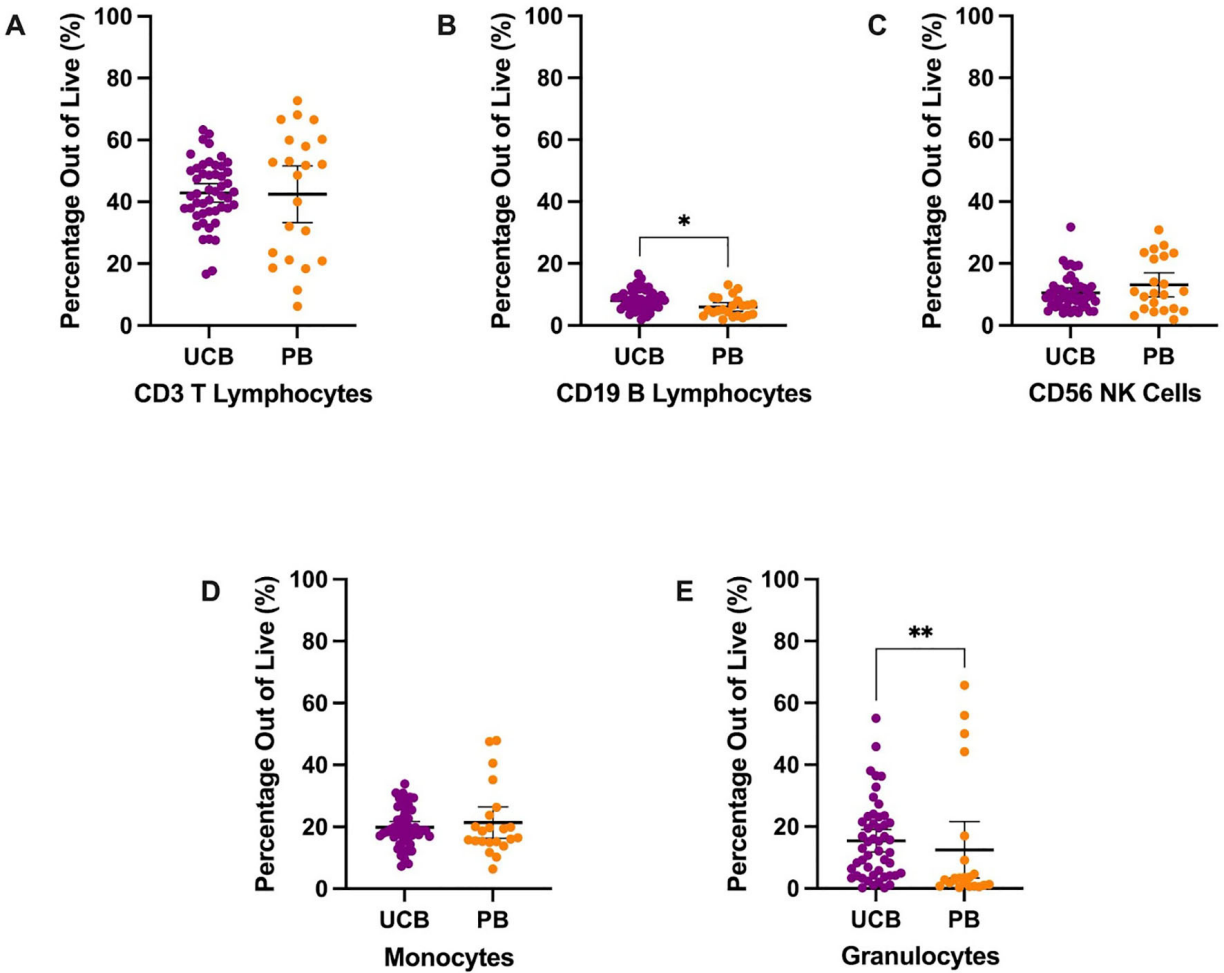
Overall immune composition of UCB and PB samples by mean percentages with error bars reflecting the 95% confidence interval; significance noted with asterisks **(A)** CD3+ T lymphocytes **(B)** CD19+ B lymphocytes **(C)** CD56+ NK cells **(D)** CD13+ HLA-DR+ monocytes **(E)** CD13+ CD66b+ granulocytes. (*: p-value<0.05, **: p-value: <0.01).

**Figure 2:**
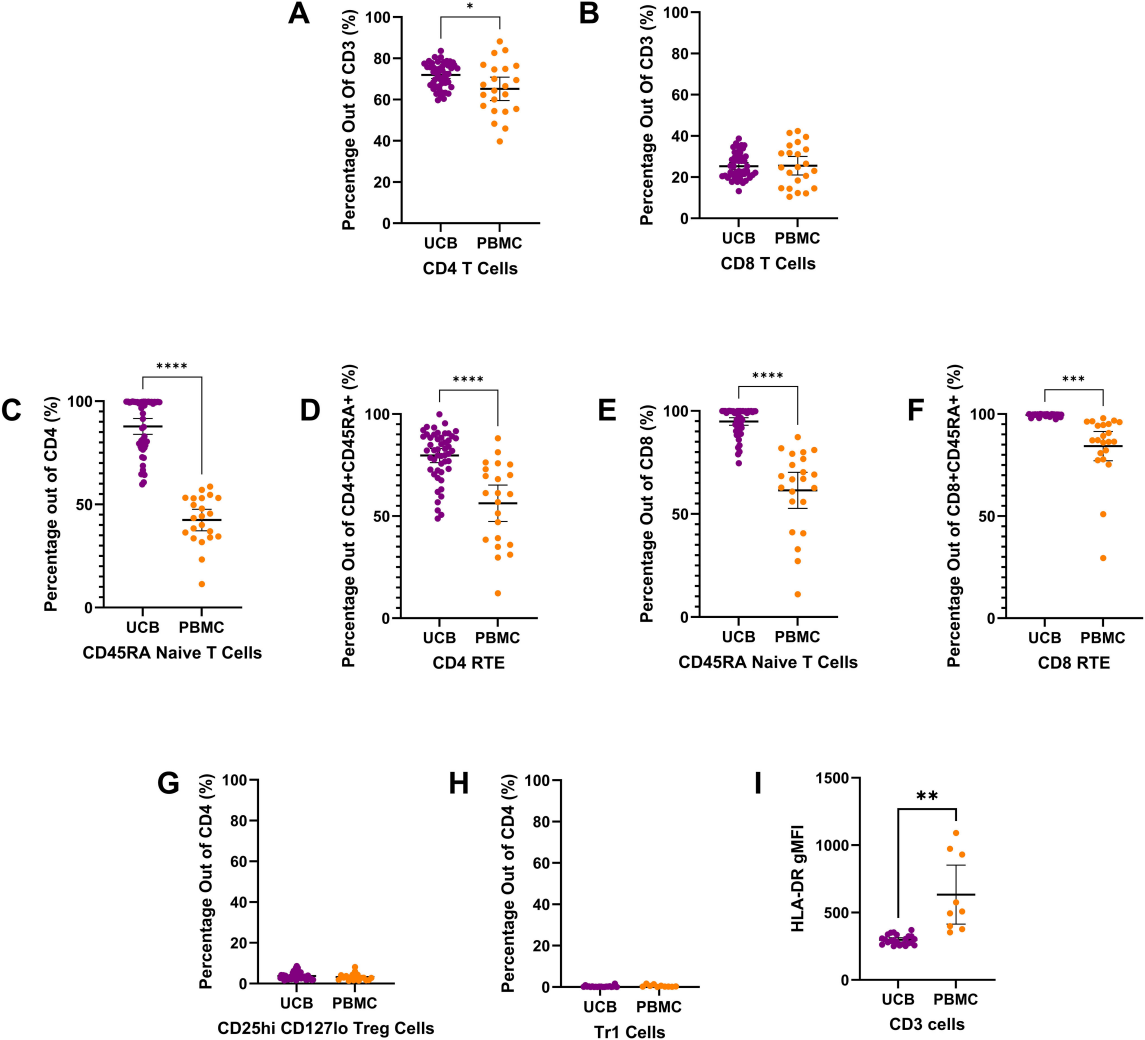
T cell subtype composition of UCB and PB samples by mean percentages with error bars reflecting the 95% confidence interval; significance noted with asterisks **(A)** CD4+ T lymphocytes **(B)** CD8+ T lymphocytes **(C)** CD4+ CD45RA+ naïve T lymphocytes **(D)** CD4+ CD45RA+ CD31+ Recent thymic emigrants **(E)** CD8+ CD45RA+ naïve T lymphocytes **(F)** CD8+ CD45RA+ CD31+ Recent thymic emigrants **(G)** CD4+ CD24hi CD127lo regulatory T cells **(H)** CD4+ CD49b+ LAG3+ type 1 regulatory T cells **(I)** Geometric mean fluorescence intensity (GMFI) of HLA-DR on CD3+ T lymphocytes. (*: p-value<0.05, **: p-value:<0.01 ***: p-value≤0.001 ****: p-value<0.0001).

**Figure 3:**
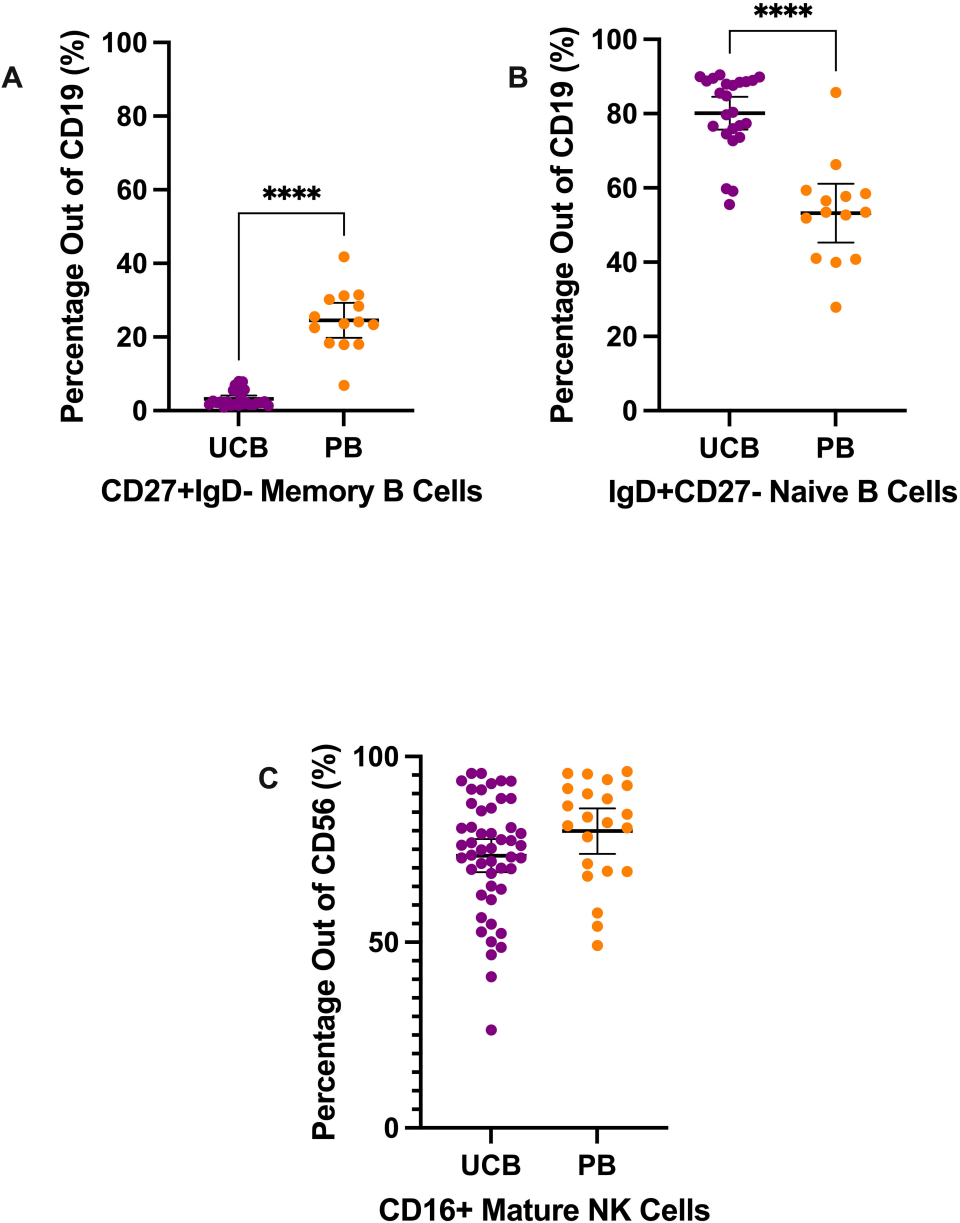
B cell and NK cell subtypes composition of UCB and PB samples by mean percentages with error bars reflecting the 95% confidence interval; significance noted with asterisks **(A)** CD19+ CD27+ IgD- memory B lymphocytes **(B)** CD19+ IgD+ CD27− naïve B lymphocytes **(C)** CD56+ CD16+ mature NK cells. (****: p-value<0.0001).

Following MNC isolation, 1 million cells per sample were stained with antibodies against markers at optimal concentrations according to manufacturer protocol (**Supplementary Table 1**). At least 100,000 events were acquired on a Beckman Coulter CytoFLEX flow cytometer and analyzed using FlowJo software (version 10.8.1) (**Supplementary Figure 1**). The study began with two panels to capture broad immune MNC subsets. They were updated to include new markers and a third panel to identify naive B cells (IgD), monocytes and granulocytes (CD66b), and TR1 cells (CD19b, LAG3). While our flow dataset has a total of 22 PB donors and 50 UCB donors, **Figures 1D, E**, **2H**, **3A, B**, have 14 PB donors and 24 UCB donors, reflecting the donors sampled after the panels were updated.

### Single cell RNA-seq library preparation and sequencing

Libraries were prepared using Chromium Next GEM Single Cell 3′ Reagent Kits v3.1 single index kit according to the manufacturer’s protocol (10x Genomics), targeting 10,000 cells per sample. 12 cycles of cDNA amplification were done for all samples. Individual libraries were quality checked on an Agilent 4200 Tapestation 827 using D5000 screen tape. Next, KAPA library quantification kit (#KK4923) was used for qPCR on a BioRad CFX96 RT PCR thermal cycler. Single index libraries were sequenced on.

### QC of RNA-seq data

**Figure 4:**
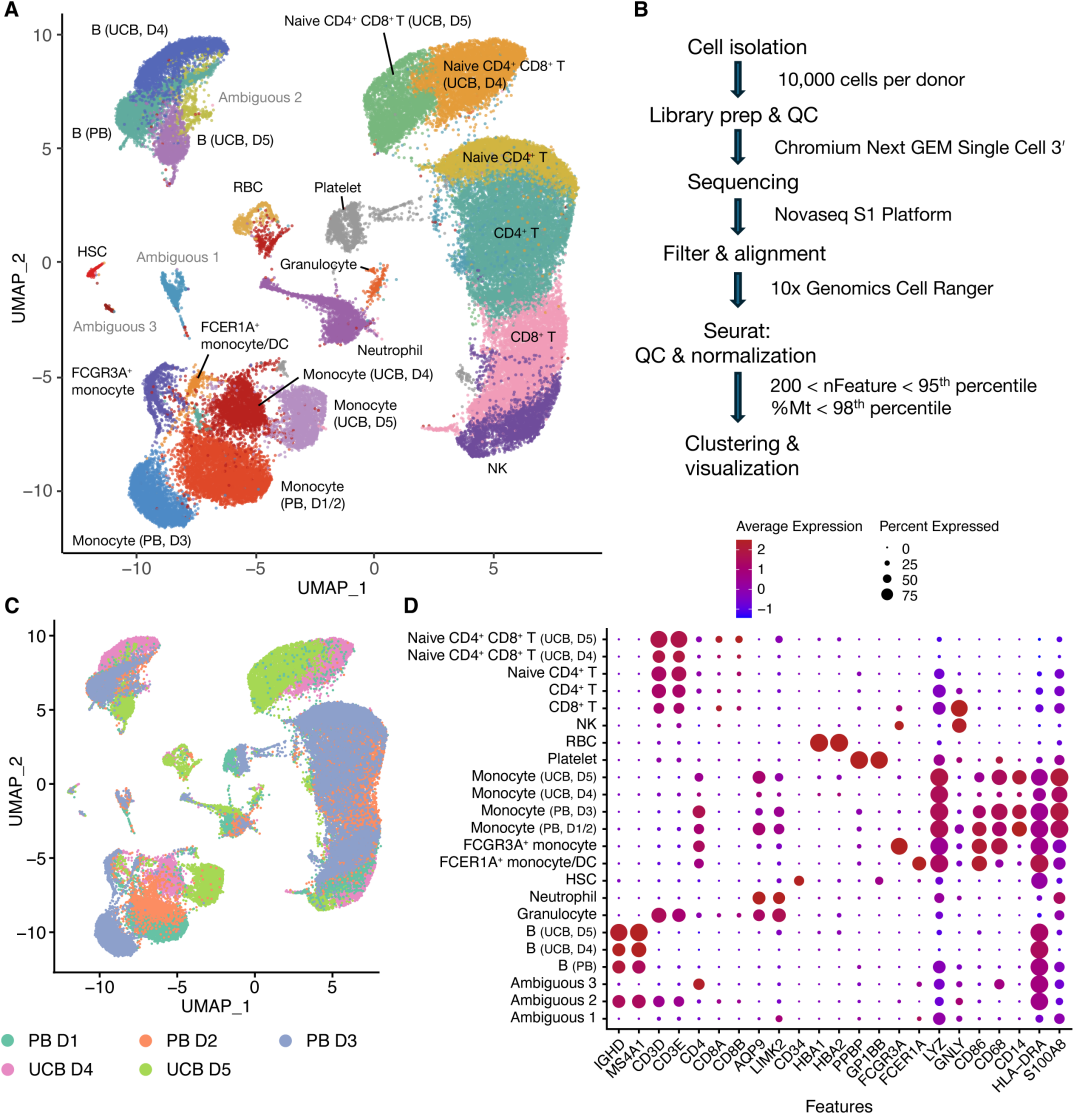
RNA-seq data showing cell clusters from UCB and PB donors **(A)** UMAP plot of clustered PB (n=3) and UCB (n=2) donor cells. D stands for donor. **(B)** Flow chart of analysis **(C)** UMAP plot of clustered PB and UCB donor cells by donor contribution **(D)** Dotplot of cell clusters based on lineage-specifying genes.

10x Genomics Cell Ranger was used to filter and align FASTQ files. Data analysis was performed in an R environment with Seurat (16) (https://satijalab.org/seurat/). Data were first filtered for quality based on number of unique features as well as mitochondrial counts. QC criteria were 200<nFeature<95th percentile and % Mitochondrial reads<98th percentile per cell for each donor. Log-normalization was performed on each cell to normalize feature expression measurements by total expression. These were performed according to donor type (UCB or PB) separately (**Supplementary Table 4**). The two Seurat objects were then merged for scaling and clustering (**Figure 4B**). Cell clusters were labeled based on lineage specifying genes (**Supplementary Table 5**) and clusters that were attributed to a single donor were specified.

### Clustering and labeling of RNA-seq data

Clustering in Seurat is based on finding a subset of features exhibiting high variability in expression in the dataset. These were identified using the ‘VariableFeatures’ function. The counts were then scaled before performing dimensional reduction using Principal Component Analysis (PCA). We then used Uniform Manifold Approximation and Projection (UMAP) to project cells in two dimensions, with similar cells being plotted closer together. Known hematopoietic lineage markers were projected onto cell populations using ‘FeaturePlot’ to identify hematopoietic and immune cell types that could be compared with flow cytometry data. Elbow plots reflecting the standard deviations of the principle components were used to identify the number of significant dimensions that were used for clustering.

### Statistics

Statistical analysis was performed by GraphPad Prism (Version 9.4.0) and SAS^®^ Studio (Release 3.81; Enterprise Edition). Data reported in the figures reflect the mean with a 95% Confidence Interval and we report mean and Standard Deviation (SD) in the text, assessing the heterogeneity within UCB MNCs samples and their specific immune subtypes. The relationship between UCB and PB MNCs was assessed using a parametric unpaired t-test with Welch’s correction or a nonparametric Mann-Whitney test based on Shapiro-Wilk normality test results. Detailed statistical results can be found in **Supplementary Tables 2, 3**. P values reported are as follows: *: p-value<0.05, **: p-value:<0.01 ***: p-value=<0.001 ****: p-value<0.0001.

## Results

### Umbilical cord blood results

**Figure 5:**
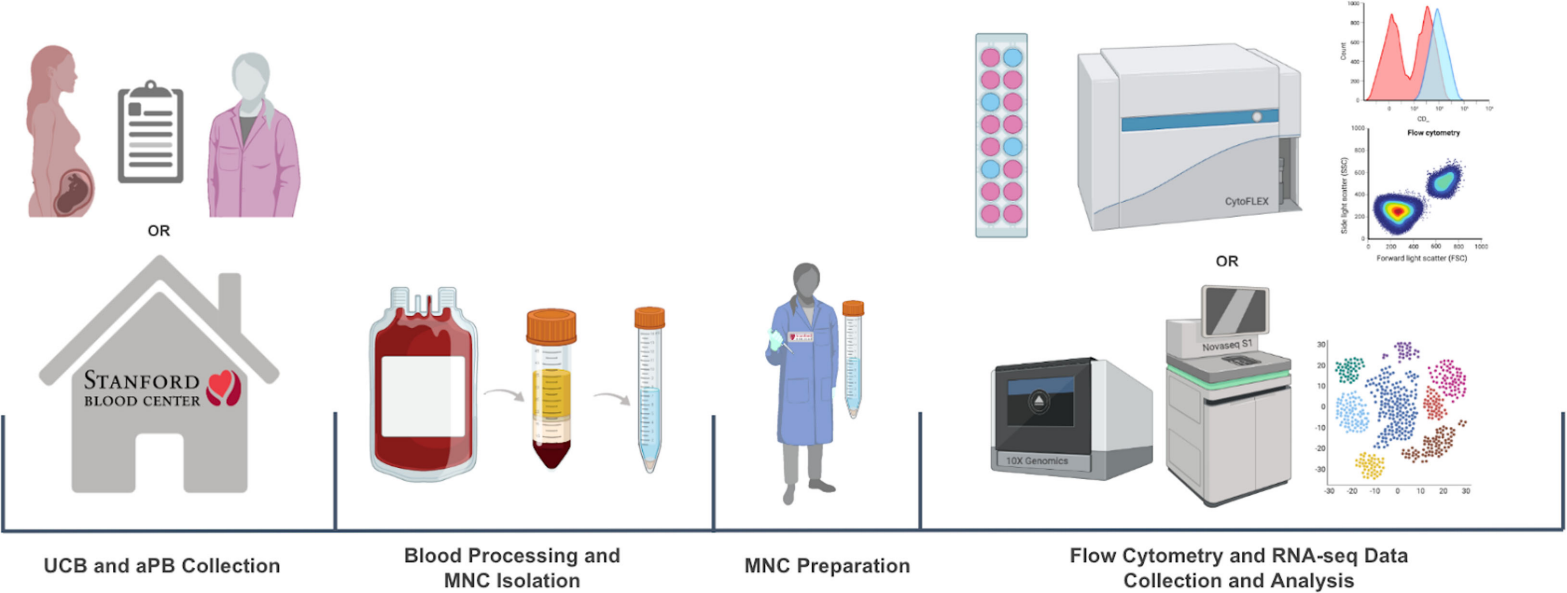
Umbilical cord blood (UCB) and adult peripheral blood (PB) workflow.

UCB was obtained through the collaboration between the Binns Program for Cord Blood Research and Lucile Packard Children’s Hospital. PB from healthy adults was obtained through the Stanford Blood Center. The collected whole blood samples were then processed through density gradient separation to isolate MNCs. For flow cytometry, MNCs were stained with antibodies against immunologic markers of interest, and data was collected with CytoFLEX and analyzed with FlowJo. For RNA-seq, the samples went through library prep (10x genomics), QC, sequencing (Novaseq S1), and analyzed with Seurat (**Figure 5**).

### Flow cytometry immune cell population characterization

MNCs were stained and analyzed to describe the populations’ major immune subpopulations, including T lymphocytes, monocytes, granulocytes, natural killer (NK) cells, and B lymphocytes (**Table 1**, **Figure 1**). The largest mean proportion of MNCs in UCB in Panel 1 was identified as T lymphocytes (CD3+) at an average of 42.89% (SD: 10.37) of the live cells (**Figure 1A**). The smallest proportion of MNCs in UCB was, on average, 8.07% (SD: 3.17) B lymphocytes (CD19+) (**Figure 1B**). UCB composition was, on average, 19.85% monocytes (SD: 6.38) and 10.57% NK cells (SD: 5.44) (**Figures 1C, D**). Additionally, we found UCB to have an average of 15.40% granulocytes (SD: 12.67) (**Figure 1E**). The remainder of the UCB and PB descriptive statistics can be found in **Supplementary Table 2**. The immune composition of UCB compared to PB did not significantly differ for T lymphocytes, NK cells, monocytes, and granulocytes (p>0.05). However, UCB had a 2.13% higher mean proportion of B lymphocytes compared to PB (p=0.0125) (**Supplementary Table C**).

**Table 1:** Cell population and specificity.

LINEAGE	SPECIFICITY
CD3+	T Lymphocytes
CD3+ CD4+	CD4 T Cells
CD3+ CD8+	CD8 T Cells
CD3+ CD45RA+	Naïve T Cells
CD3+ CD45RA+ CD31+	Recent Thymic Emigrants (RTE)
CD3+ CD4+ CD25hi CD127lo	Regulatory T Cells
CD3+ CD4+ CD49b+ LAG3+	Tr1
CD13+ HLA-DR+ CD66b-	Monocytes
CD13+ HLA-DR- CD66b+	Granulocytes
CD19+	B Lymphocytes
CD3- CD19- CD56+	NK Cells
CD3- CD19- CD56+ CD16+	Mature NK Cells
CD19+ IgD+ CD27-	Naïve B Cells
CD19+ CD27+ IgD-	Mature B Cells

CD3+ T lymphocyte populations (**Figure 2**) make up a large percentage of the live mononuclear cells seen in umbilical cord blood and adult peripheral blood. In UCB and PB, the T lymphocyte population is mostly composed of CD4 T cells and CD8 T cells (**Figure 2**). On average, the T lymphocyte cells of UCB were 71.95% CD4 T cells (SD: 6.03) and 25.33% CD8 T cells (SD: 6.04) (**Figures 2A, B**). UCB had a mean CD4 T cell proportion that was 6.73% higher than the proportion for PB (p=0.0265) (**Supplementary Table 3**). There was no significant difference in CD8 T cells between UCB and PB (p>0.05) (**Supplementary Table 3**).

Looking closer at the subtypes of CD4 T cells, a large proportion were CD4 CD45RA naive T cells in UCB. Out of CD4 T cells, 87.79% were CD4 naive T cells (SD: 13.35), (**Figure 2C**). UCB had a mean CD4 naive T cell count that was double the mean count in PB (p<0.0001) (**Supplementary Table 3**). Within the CD4+ naive T cell population, there was also a significantly higher average cell count of recent thymic emigrants (RTE) cells in UCB compared to PB (p<0.0001) (**Supplementary Table 3**; **Figure 2D**). UCB had an average of 79.67% RTE subpopulation (SD: 12.14) within the CD4 CD45RA naive T cells. Comparatively, the mean percentage of RTE cells within the CD4 naive T cells in the PB samples was 23.39% lower (**Supplementary Table 3**).

From the CD4 T cells, there were also small subpopulations of Regulatory T (Treg) and Type 1 regulatory cells (TR1) in both UCB and PB (**Figures 2G, H**). The UCB samples had an average of 3.71% (SD: 1.69) and 0.27% (SD: 0.37) from the CD4 T cell population identified as Treg (**Figure 2G**) and TR1 cells (**Figure 2H**), respectively. No significant difference was found in the mean subpopulation percentages for TR1 or Treg cells between UCB and PB (p>0.20) (**Supplementary Table 3**). The geometric mean fluorescence intensity of HLA-DR on CD3 T cells was significantly lower on UCB cells compared to PBMCs (p=0.077).

When analyzing the subpopulations of CD8 T cells, the majority were CD8 CD45RA naive T cells in UCB and PB (**Figure 2E**). On average, CD8 T cells in UCB had 94.87% (SD: 6.52) cells identified as CD45RA naive T cells. Similar to the CD4 naive T cells, UCB had a 33.39% higher proportion of the CD8 T cells as naive compared to PB (p<0.0001) (**Supplementary Table 3**). Of these CD8 CD45RA naive T cells, an average of 99.53% (SD: 0.55) were recent thymic emigrants (RTE) in UCB (**Figure 2F**). There was a 15.23% higher proportion of RTE cells in UCB CD8 naive T cells compared to PB CD8 naive T cells (p=0.0002) (**Supplementary Table 3**).

Mononuclear cells were also stained and analyzed to characterize natural killer (NK) cells and B cells (**Figure 3**). Mature NK cells were gated from the CD3- CD19- CD56+ population (**Supplementary Figure 1**) (6, 17). On average, 73.32% of the CD56+ cells (SD: 15.55) were mature NK cells in the UCB samples (**Figure 3C**). The difference in percentage of mature NK cells between UCB and PB samples was not significant (p>0.05) (**Supplementary Table 3**).

Lastly, we analyzed the mature vs naive subtypes of B lymphocytes (**Figures 3A, B**). From the CD19+ B lymphocyte population, UCB had a dramatically larger mean proportion of naive B cells compared to its mean proportion of memory B cells—80.12% (SD: 10.40) versus 3.19% (SD: 2.23), respectively (**Figures 3A, B**). Meanwhile, the B lymphocytes in PB samples were, on average, 53.24% naive B cells (SD: 13.71) and 24.54% memory B cells (SD: 8.22) (**Figures 3A, B**). From their B lymphocyte populations, UCB had a mean proportion of memory B cells nearly eight times smaller compared to PB (p<0.0001) (**Supplementary Table 3**). Additionally, the proportion of naive B cells from the B lymphocytes was an average of 26.88% higher in UCB than in PB (p<0.0001) (**Supplementary Table 3**).

### sc-RNAseq results

#### Combined UCB-PB scRNA-seq clustering

After library prep and sequencing, read alignment was performed with 10x Genomics Cell Ranger 7.0.1 using 10x Genomics Cloud Analysis (**Figure 4B**). One UCB donor was excluded from analysis because Cell Ranger detected an estimated number of 111,851 cells when only 10,000 cells were used for library prep. For the donor samples included in the analysis, we proceeded to data processing using Seurat. Cells were filtered based on having between 200 and the 95th percentile of nFeatures, as well as less than the 98th percentile of percentage mitochondrial reads per donor (**Figure 4B**). After QC, an average of 92.8 ± 0.4% of cells were retained (**Supplementary Table 4**) across all donor samples. We obtained 36,915 cells from PB donors (n=3 donors) and 21,728 cells from UCB donors (n=2 donors). The normalized UCB and PB scRNA-seq datasets were merged to create a combined dataset of 58,643 cells (**Figure 4A**). Clustering analysis separated cells into 25 clusters which were annotated based on lineage-specifying genes (**Figures 4A, C, D** and **Supplementary Figure 2**) from the literature (2, 16, 18, 19). Clusters without a clear cell lineage were labeled ambiguous.

### Concordance between flow cytometry and scRNA-seq data

**Figure 6:**
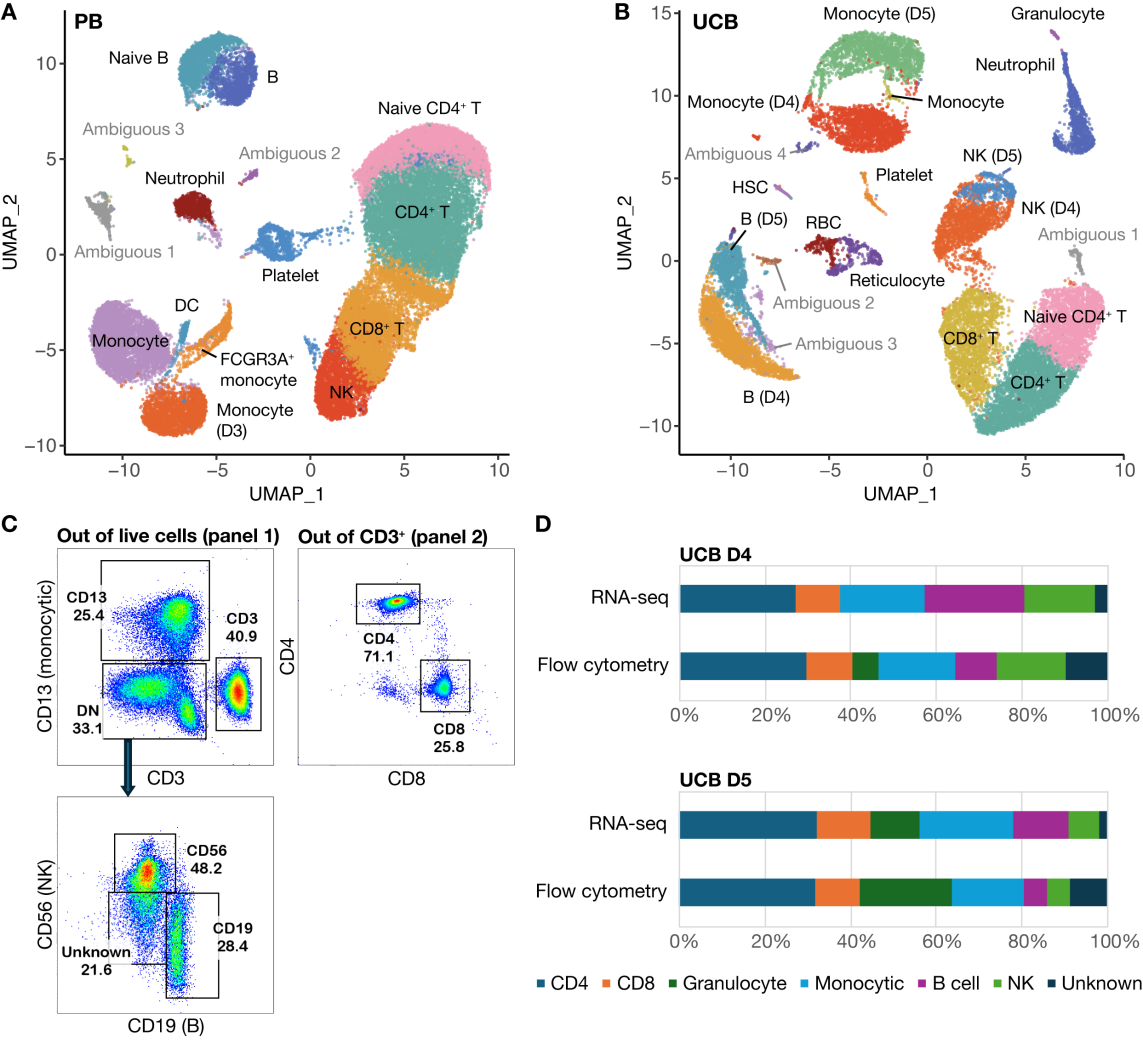
scRNA-seq data with cells clustered according to donor type. **(A)** UMAP of clustered PB donor cells (n=3) **(B)** UMAP of clustered UCB donor cells (n=2) **(C)** Gating strategy for cell types identified by flow analysis **(D)** Comparative cell composition from flow cytometry versus scRNA-seq data.

In addition to analyzing the merged datasets, we clustered the UCB and PB data separately resulting in 15 clusters for PB donors (**Figure 6A**) and 20 clusters for UCB donors (**Figure 6B**). These were annotated based on lineage-specifying genes (**Supplementary Figure 3** and **Supplementary Table 5**); clusters without a clear cell lineage were labeled ambiguous. The cell type proportions derived from our scRNA-seq analysis for the two UCB donors aligned well with the proportions derived from our flow cytometry data (**Figure 6D**, **Table 2**). Cell identities were obtained from flow through the markers CD13 (Monocytic), CD56 (NK), CD19 (B cell) and CD3 (T lymphocyte) from Panel 1. The proportion of CD4+ and CD8+ cells out of total CD3+ cells were obtained from Panel 2 out of total CD3 (**Figure 6C**). The data from flow cytometry contained more cells with an unknown identity because of the limited number of markers we could use to classify them. Both datasets showed a smaller proportion of B and NK cells, but a larger proportion of Granulocytes in UCB D5 compared to UCB D4. For example, UCB donor 5 had a smaller proportion of NK cells in both datasets (5.6% by flow, 7.1% by scRNA-seq) compared to UCB donor 4 (16.2% by flow, 16.7% by scRNA-seq) (**Figure 6D**, **Table 2**). The proportions of CD4, CD8 and NK cells were most similar across datasets. Using the Chi-squared test, we did not detect a significant difference in the cell type proportions identified by flow and scRNA-seq in both UCB donor 4 (χ^2^ = 0.152, df=6, p=0.999) and UCB donor 5 (χ^2^ = 0.128, df=6, p=0.999).

**Table 2:** Cell-type proportions from UCB MNC data from flow cytometry and scRNA-seq.

Sample	CD4	CD8	Granulocyte	Monocytic	B cell	NK	Unknown
UCB D4 flow	29.5%	10.7%	6.0%	18.4%	9.5%	16.2%	9.6%
UCB D4 RNA-seq	27.2%	10.5%	0.0%	19.6%	23.2%	16.7%	2.8%
UCB D5 flow	31.9%	10.4%	21.6%	16.7%	5.2%	5.6%	8.6%
UCB D5 RNA-seq	32.2%	12.6%	11.4%	21.8%	13.1%	7.1%	1.8%

## Discussion

The overall UCB MNC composition out of live cells consisted of T- and B-lymphocytes, natural killer (NK) cells, monocytes and granulocytes (**Figure 1**). UCB had a significantly higher percentage of CD19+ B-lymphocytes compared to adult PB (p=0.0125). There were no significant differences between the other cell populations. Based on range, UCB T-lymphocytes and granulocytes were the most heterogeneous, with the greatest inter-donor variability. We observed that most UCB donors had a high number of CD66b+ granulocytes compared to PB donors (**Figure 1E**). While elevated plasma G-CSF and neutrophil counts in UCB (20) have been previously reported, we were surprised to observe the presence of granulocytes since we were studying the MNC fraction, which should exclude polymorphonuclear cells. While Low-density Granulocytes (LDGs) found in the PB fraction have been reported in systemic lupus erythematosus (SLE) and other systemic autoimmune and autoinflammatory diseases (21), their role has not been studied extensively in healthy individuals or UCB. While we saw that most UCB donors had more granulocytes (**Figure 1E**), the trend was not significant due to a few outlier PB donors which could have underlying, inflammatory conditions.

UCB CD3+ T-lymphocytes were further characterized by CD4+ and CD8+ immunophenotyping, with a higher number of CD4+ subtypes (**Figure 2**). UCB had a higher percentage of CD3+CD4+ T-cells compared to PB (p=0.0265). Immaturity was determined by CD3+CD45RA+, detecting naive T-cell populations, and further by CD3+CD45RA+CD31+, detecting Recent Thymic Emigrants (RTE). Both subtypes were significantly higher among UCB samples compared to PB samples for both CD3+CD4+ and CD3+CD8+ populations, confirming the relatively immature properties of T-lymphocytes in human UCB. Moreover, HLA-DR expression on CD3 cells in UCB is significantly lower than in adult PBMCs (**Figure 2I**). In an allogeneic setting, the lowered antigen presentation could reduce the host immune response against these cells. These could explain why UCB transplant is associated with a lower incidence of graft-versus-host disease (GVHD) in the clinical setting compared to bone marrow stem cell transplantation (4). The characteristics of UCB cells described above support the use of UCB T cells for allogeneic T cell therapy, including chimeric antigen receptor therapy (22–27).

CD19+ B-lymphocytes were composed of a higher number of naive or transitional B cells versus memory B cells (**Figure 3**) (28). There was a significantly higher percentage of CD19+CD27+IgD- in PB compared to UCB, and a significantly higher percentage of CD19+IgD+CD27- in UCB compared to PB, again confirming the relatively immature properties of human UCB. Other studies have shown that although UCB-derived B cells have a more naive phenotype, they have a distinct transcriptional program conferring accelerated responsiveness to stimulation and facilitated IgA class switching (29). Mature NK cell composition was not significantly different between UCB and PB. Similarly, Treg and TR1 CD3+CD4+ composition in UCB samples were not significantly higher compared to PB, suggesting that neonatal tolerance is largely mediated by the maternal immune system in keeping with the literature (30–32).

To our knowledge, this study of the immune composition of 50 UCB donors is the largest to date. The findings from flow cytometry were further supported by scRNA-seq data which showed concordance between flow cytometry and scRNA-seq data. While cells clustered into expected hematopoietic lineages observed in the literature (13, 33, 34), our results highlight that cells from PB donors tended to cluster together while cells from UCB donors tended to cluster separately (**Figure 4C**). There are separate clusters for UCB B cells, naive T cells, and Monocytes. In the PB dataset, 17 out of 18 clusters contained cells from multiple donors while in the UCB dataset, more clusters contained cells from an individual donor (6 out of 19 clusters). This suggests that while there may be variation at birth, cell types tend to converge in adulthood as the immune system matures. Another surprising observation is that naïve CD3 T cells from UCB cluster together regardless of whether they are CD4 or CD8 (**Figure 4A**). Our flow data support that the overwhelming majority of CD3 cells from UCB donors are naive: 87.79% of CD4 T cells and 94.87% of CD8 T cells are CD45RA+. These UCB T cells cluster separately from adult T cells, which in contrast form more differentiated clusters of CD4 naïve, CD4, and CD8 T cells. This adds to our understanding of adaptive immune maturation where T-Cell Receptor (TCR) stimulation in different immune and tissue contexts causes more differentiated but stereotyped effector cell types to emerge.

While we did not explore this directly, there has been a significant body of work on understanding how immune cells differentiate and persist in the body (35). Studies in humans and rhesus macaques have led to the identification of CD8 stem cell memory T cells that have enhanced capacity for self-renewal and multipotent ability to derive other memory and effector subsets (36, 37). Interestingly, these cells also express the naive marker CD45RA. Additional markers and molecular regulators of these cells have been described and include BACH2 and TCF7 (36, 38–42). Since these stem-like CD8 cells are derived from exposure to a particular immune context (antigens, other immune cells and the cytokine milieu) in adults, they may not naturally be found in naive CD45RA+ UCB cells. Nevertheless, efforts to characterize and differentiate memory or stem-like T cell subsets (43) from UCB immune cells could enable the development of more potent and long-lasting immune cell therapies.

There are important considerations when interpreting the data from our study. First, our UCB samples were obtained from pregnant individuals at Lucile Packard Children’s Hospital (LPCH), so donor samples reflect the diverse ethnic backgrounds found in the Bay Area. LPCH serves insured, uninsured and underinsured families, and the patient population is largely Hispanic (56%), followed by Pacific Islander (13%), African American (11%), Asian (8%), Caucasian (7%) and unspecified (5%) (44). Additionally, we obtained the samples through the Binns Program for Cord Blood Research and the Stanford Blood Center, so conditions such as autoimmunity and neoplasms were screened and the patients consented were generally healthy (14). These factors influence the generalizability of our data. Notably, the health screening process might not have included specific conditions. Because our samples are de-identified, we cannot analyze certain specific findings, or make correlations of specific cell characteristics with specific medical histories.

One limitation of the study is the inability to capture all immune subsets. Since our aim was to study the MNC fraction from donor samples, polymorphonuclear cells in the densest fraction pelleted after Ficoll centrifugation were excluded. While we observed LDGs in the MNC fraction, the differences between PB and UCB were not statistically significant and may be less robust because we know less about the health status of the PB donors. These cells, the majority of which are Neutrophils, are sensitive to slight changes in temperature or ion concentrations (45), and could have been activated and lost during flow analysis or library preparation for RNA-seq. Notably, the Granulocyte population was present in the flow data of UCB donor 4 but absent from the scRNA-seq dataset (**Table 2**). While well-characterized in autoimmune conditions, the LDG population warrants further study in UCB and healthy individuals.

Human UCB is an overlooked resource and a material that is often discarded after delivery but may hold therapeutic potential. L. Buzaníska et al. (2002) used the CD34-negative fraction of human UCB to obtain neural-like stem cells, demonstrating the high self-renewal potency of these populations (46). UCB derived regulatory T cells (Tregs) have also been investigated clinically as a source of cells for adoptive Treg transfer to prevent GVHD (47). Upon isolation, they have been shown to have comparable potency to adult peripheral blood (PB) derived Tregs (48) and to contain a higher proportion of naïve cells (CD45RA+), which have longer-term phenotypic and epigenetic stability as compared to memory Tregs (49). We were able to study this cell source in-depth through the Binns Program for Cord Blood Research. Our study provides a robust characterization of the immune subset composition of a large cohort of human umbilical cord blood and control adult peripheral blood donors through flow cytometry immunophenotyping. We were able to combine this with finer grained scRNA-seq analysis which showed concordance in the proportion of immune cell types but captured more differentiated subtypes of cells. These analyses yield the intriguing possibility that immune heterogeneity of individuals at birth gives way to more stereotyped immune subsets as the immune system is exposed to the external environment and undergoes maturation.

## Data Availability

The data presented in the study are deposited in the gene expression omnibus (https://www.ncbi.nlm.nih.gov/geo/) and can be accessed by the accession number, GSE302276.
