# Supplementary material for: Immune composition of the mononuclear cell fraction of human umbilical cord blood
Source: Front Immunol. 2025 Aug 1;16:1614230. doi: 10.3389/fimmu.2025.1614230 (PMC12354479; doi:10.3389/fimmu.2025.1614230)
Supplement: Supplementary file 1 [file DataSheet1.pdf]

Supplemental Tables

Supplemental Table 1. Panel design (Beckman Coulter CytoFLEX Flow Cytometer). (A) Initial UCB and aPB samples were processed with Original Panels. Later revision prompted changes to Panels 1 and 2 and addition of Panel 3 for a more comprehensive analysis of cell types. (B) Clone and catalog number for antibodies used.

1A

MARKER	FLUOROPHORE
Live/Dead	Ghost Dye BV510
CD3	PE-CF594
CD13	PE
CD14	BV421
CD19	BV605
CD56	PE-Cy7
HLA-DR	FITC
CD27	APC-Cy7
CD16	APC

Original Panel 1 (General)

MARKER	FLUOROPHORE
Live/Dead	Ghost Dye BV510
CD3	PE-CF594
CD4	BV421
CD8	BV605
CD25	APC-Cy7
CD127	FITC
CD45RA	APC
CD31	PE-Cy7

Original Panel 2 (T Cell)

MARKER	FLUOROPHORE
CD3	PE-CF594
CD13	PE
CD14	BV421
CD19	BV605
CD56	PE-Cy7
HLA-DR	FITC
CD66b	APC-Cy7
CD16	APC
Live-Dead	Ghost Dye BV510

Updated Panel 1 (General)

MARKER	FLUOROPHORE
CD3	PE-CF594
CD4	BV421
CD8	BV605
CD31	PE-Cy7
CD45RA	APC
CD19	BV650
IgD	FITC
CD27	APC-Cy7
Live-Dead	Ghost Dye BV510
CD14	BV510 (dump)

Updated Panel 2 (T & B Lymphocytes)

MARKER	FLUOROPHORE
CD3	PE-CF594
CD4	APC Cy7
CD25	BV605
CD127	BV421
CD49b	FITC
LAG3	APC
Live-Dead	Ghost Dye BV510
CD14	BV510 (dump)

Panel 3 (Regulatory T Lymphocytes)

1B

MARKER	FLUOROPHORE	CLONE	CATALOG NUMBER
Live/Dead	Ghost Dye BV510	-	50-201-4118
CD3	PE-CF594	UCHT1	BDB562280
CD13	PE	WM15	BDB560998
CD14	BV421	MφP9	BDB563744
CD19	BV605	SJ25C1	BDB562654
CD56	PE-Cy7	B159	BDB557747
HLA-DR	FITC	LN3	50-112-2195
CD27	APC-Cy7	O323	50-165-906
CD16	APC	3G8	BDB561248
CD4	BV421	SK3	BDB566907
CD8	BV605	SK1	BDB564115
CD25	APC-Cy7	M-A251	BDB561782
CD127	FITC	HIL-7R-M21	BDB560549
CD45RA	APC	5H9	BDB561210
CD31	PE-Cy7	WM59	BDB563651
CD66b	APC-Cy7	G10F5	305125
IgD	FITC	IA6-2	50-213-1878
CD14	BV510 (dump)	MφP9	BDB563079
CD4	APC Cy7	RPA-T4	BDB557871
CD25	BV605	BC96	BDB567572
CD127	BV421	hIL-7R-M21	BDB562436
CD49b	FITC	REA188	130-100-335
LAG3	APC	REA351	130-119-567

Supplemental Table 2. Detailed statistics for MNC and PBMC comparisons.

Pop	N Obs	Variable	Mean	Std Dev	Lower 95% CL for Mean	Upper 95% CL for Mean	N	t Value	Pr > |t|
CD3_live	49	UCB	42.888	10.369	39.91	45.867	49	28.95	<.0001
		PB	42.368	20.727	33.178	51.558	22	9.59	<.0001
CD19_live	49	UCB	8.07	3.168	7.16	8.98	49	17.83	<.0001
		PB	5.836	3.181	4.426	7.247	22	8.61	<.0001
CD56_live	49	UCB	10.573	5.439	9.01	12.135	49	13.61	<.0001
		PB	12.95	8.883	9.012	16.888	22	6.84	<.0001
Monocytes_live	49	UCB	19.852	6.375	18.021	21.683	49	21.8	<.0001
		PB	21.386	11.676	16.071	26.701	21	8.39	<.0001
Granulocytes_live.	49	UCB	15.402	12.667	11.763	19.04	49	8.51	<.0001
		PB	12.386	20.668	3.223	21.55	22	2.81	0.0105
CD4_CD3	50	UCB	71.953	6.029	70.24	73.667	50	84.39	<.0001
		PB	65.132	12.749	59.479	70.784	22	23.96	<.0001
CD8_CD3	50	UCB	25.331	6.04	23.615	27.048	50	29.65	<.0001
		PBMC	25.445	10.182	20.931	29.96	22	11.72	<.0001

CD45RA_CD4.	50	UCB	87.794	13.354	83.999	91.589	50	46.49	<.0001
		PB	42.373	11.784	37.148	47.597	22	16.87	<.0001
CD45RA_CD8	50	UCB	94.869	6.522	93.015	96.722	50	102.85	<.0001
		PB	61.4	19.731	52.652	70.148	22	14.6	<.0001
CD31_Naive CD4.	50	UCB	79.671	12.141	76.22	83.122	50	46.4	<.0001
		PB	56.164	20.084	47.259	65.068	22	13.12	<.0001
CD31_Naive CD8	50	UCB	99.529	0.555	99.371	99.686	50	1268.63	<.0001
		PB	84.227	16.175	77.056	91.399	22	24.42	<.0001
Treg_CD4	46	UCB	3.706	1.69	3.204	4.208	46	14.87	<.0001
		PBMC	3.139	1.758	2.264	4.013	18	7.57	<.0001
Tr1_CD4	20	UCB	0.269	0.376	0.093	0.445	20	3.2	0.0047
		PB	0.47	0.583	0.053	0.887	10	2.55	0.0313
Memory B _ CD19	24	UCB	3.189	2.229	2.248	4.13	24	7.01	<.0001
		PB	24.486	8.223	19.738	29.234	14	11.14	<.0001
Naive B _ CD19	24	In	80.121	10.402	75.729	84.514	24	37.74	<.0001
		PB	53.186	13.714	45.268	61.104	14	14.51	<.0001
CD16_CD56.	49	UCB	73.319	15.553	68.851	77.786	49	33	<.0001

		PB	79.886	13.84	73.75	86.023	22	27.07	<.0001
HLA-DR- _ GM	49	UCB	33.292	23.187	26.632	39.952	49	10.05	<.0001
		PB	22.482	29.014	9.618	35.346	22	3.63	0.0016
HLA-DR+ _ GM	49	UCB	61.114	21.952	54.808	67.419	49	19.49	<.0001
		PB	71.255	28.839	58.468	84.041	22	11.59	<.0001
CD66b+ _ HLADR-	24	UCB	91.707	20.216	83.17	100.243	24	22.22	<.0001
		PB	98.092	1.851	96.974	99.211	13	191.09	<.0001
CD66b+ _ HLADR+	24	UCB	0.658	0.71	0.358	0.958	24	4.54	0.0001
		PB	0.264	0.348	0.063	0.465	14	2.84	0.0138

Supplemental Table 3. Mean differences ± SEM of PBMC and UCB populations. Differences in mean values are calculated as PBMC-UCB means. NS: p-value not significant, *: p-value<0.05, **: p-value:<0.01 ***: p-value=<0.001 ****: p-value<0.0001.

Pop	Difference Between Means (PB-UCB) ± SEM	95% Confidence Interval	P-Value	P-Value Summary	Significantly different (P < 0.05)?
CD3/live	-0.4598 ± 4.654	-10.03 to 9.109	0.9221	ns	No
CD19/live	-2.125 ± 0.8129	-3.767 to -0.4826	0.0125	*	Yes
CD56/live	2.525 ± 2.030	-1.629 to 6.679	0.2236	ns	No
Monocytes/live	1.516 ± 2.596	-3.809 to 6.842	0.5640	ns	No

Granulocytes/live	-2.904 ± 4.757	-12.64 to 6.835	0.5465	ns	No
CD4/CD3	-6.729 ± 2.854	-12.61 to -0.8528	0.0265	*	Yes
CD8/CD3	0.2231 ± 2.331	-4.554 to 5.000	0.9244	ns	No
CD45RA/CD4	-45.36 ± 3.143	-51.69 to -39.03	<0.0001	****	Yes
CD45RA/CD8	-33.39 ± 4.313	-42.31 to -24.47	<0.0001	****	Yes
CD31/Naive CD4	-23.39 ± 4.622	-32.86 to -13.92	<0.0001	****	Yes
CD31/Naive CD8	-15.23 ± 3.450	-22.40 to -8.051	0.0002	***	Yes
Treg/CD4	-0.5132 ± 0.4834	-1.500 to 0.4739	0.2968	ns	No
Tr1/CD4	0.2631 ± 0.1958	-0.1591 to 0.6854	0.2016	ns	No
Memory B / CD19	21.35 ± 2.245	16.54 to 26.16	<0.0001	****	Yes
Naive B / CD19	-26.88 ± 4.236	-35.67 to -18.10	<0.0001	****	Yes
CD16/CD56	6.617 ± 3.695	-0.8247 to 14.06	0.0800	ns	No
HLA-DR+ / GM	10.21 ± 6.896	-3.826 to 24.26	0.1482	ns	No
HLA-DR- / GM	-10.64 ± 7.038	-24.95 to 3.670	0.1399	ns	No
CD66b+ / HLADR+	-0.3311 ± 0.1731	-0.6824 to 0.02016	0.0639	ns	No
CD66b+ / HLADR-	6.578 ± 4.156	-2.006 to 15.16	0.1267	ns	No

Supplemental Table 4. Pre- and post-quality control (QC) cell estimates for each donor.

Donors	Estimated Cells (Cell Ranger)	Cell Number Post QC
PB D1	11734	10912
PB D2	12944	12036
PB D3	15022	13967
UCB D4	11877	10939
UCB D5	11635	10789
UCB D6	111851	NA

Supplemental Table 5. Cell types and their lineage specifying genes.

	**Cell Type**	**Genes**	**Naive**
1	B cell	MS4A1	IGHD
2	CD4	CD3D, CD3E, CD4	SELL, CCR7

3	CD8	CD3D, CD3E, CD8A, CD8B	SELL, CCR7
4	Neutrophil / Granulocyte	AQP9, LIMK2	
5	HSC	CD34	
6	RBC / Reticulocyte	HBA1, HBA2	
7	Platelets	PPBP, GP1BB	
8	Monocytic	LYZ, HLA-DRA, S100A8, CD86	
9	Non-classical / intermediate Monocytes	FCGR3A (CD16)	
10	DC	FCER1A	
11	NK	GNLY	

Supplemental Figures

1A

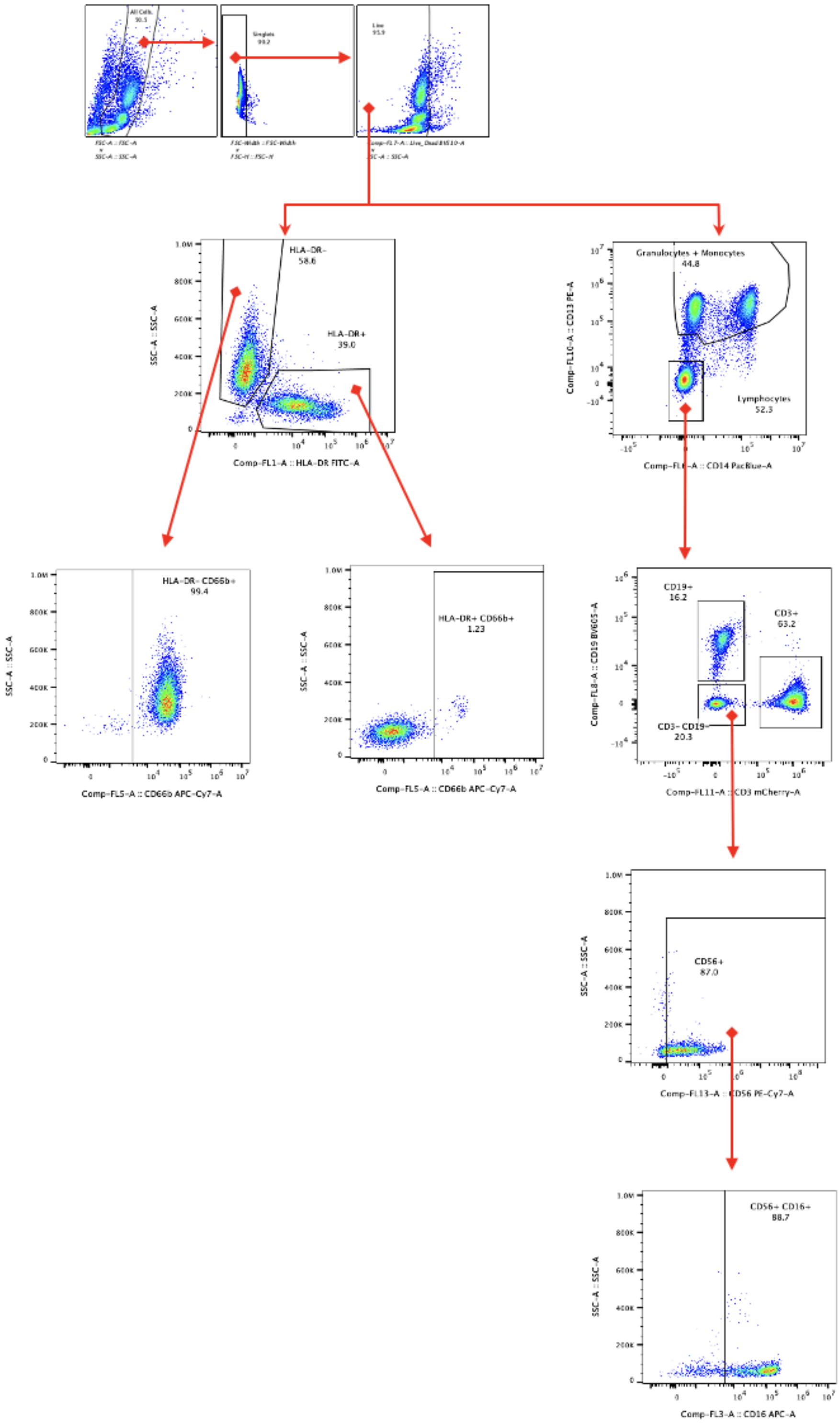
HLA-DR- 58.6
HLA-DR+ 39.0
SSC-A :: SSC-A
Comp-FL1-A :: HLA-DR FITC-A
Granulocytes + Monocytes 44.8
Lymphocytes 52.3
Comp-FL10-A :: CD13 PE-A
HLA-DR- CD66b+ 99.4
Comp-FL5-A :: CD66b APC-Cy7-A
HLA-DR+ CD66b+ 1.23
Comp-FL5-A :: CD66b APC-Cy7-A
CD19+ 16.2
CD3+ 63.2
CD3- CD19- 20.3
Comp-FL8-A :: CD19 BV605-A
Comp-FL11-A :: CD3 mCherry-A
CD56+ 87.0
Comp-FL13-A :: CD56 PE-Cy7-A
CD56+ CD16+ 88.7
Comp-FL3-A :: CD16 APC-A

1B

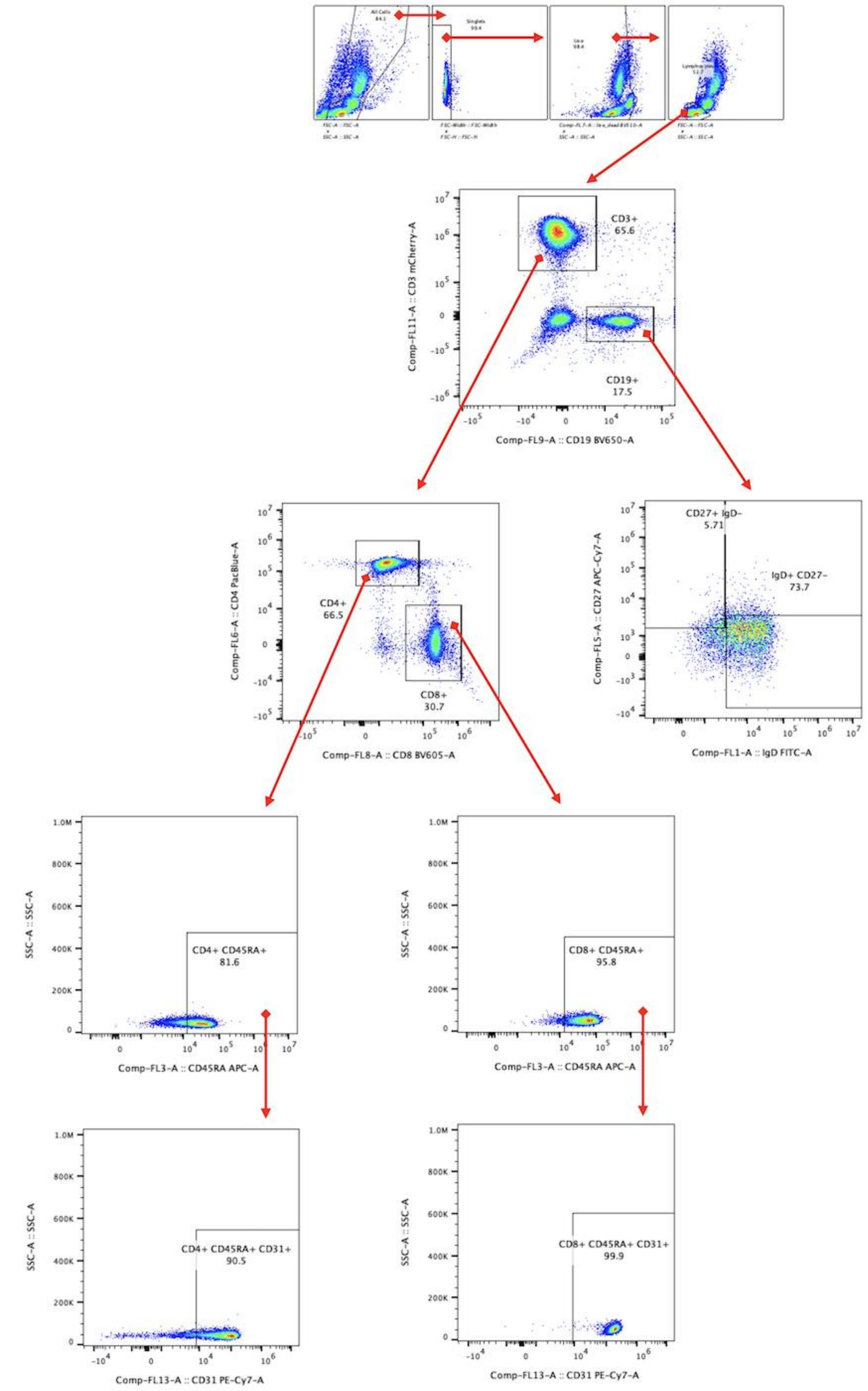

CD3+
65.6
CD19+
17.5
Comp-FL11-A :: CD3 mCherry-A
Comp-FL9-A :: CD19 BV650-A
CD4+
66.5
CD8+
30.7
Comp-FL6-A :: CD4 PacBlue-A
Comp-FL8-A :: CD8 BV605-A
CD27+ IgD-
5.71
IgD+ CD27-
73.7
Comp-FL5-A :: CD27 APC-Cy7-A
Comp-FL1-A :: IgD FITC-A
CD4+ CD45RA+
81.6
CD8+ CD45RA+
95.8
SSC-A :: SSC-A
Comp-FL3-A :: CD45RA APC-A
CD4+ CD45RA+ CD31+
90.5
CD8+ CD45RA+ CD31+
99.9
Comp-FL13-A :: CD31 PE-Cy7-A

1C

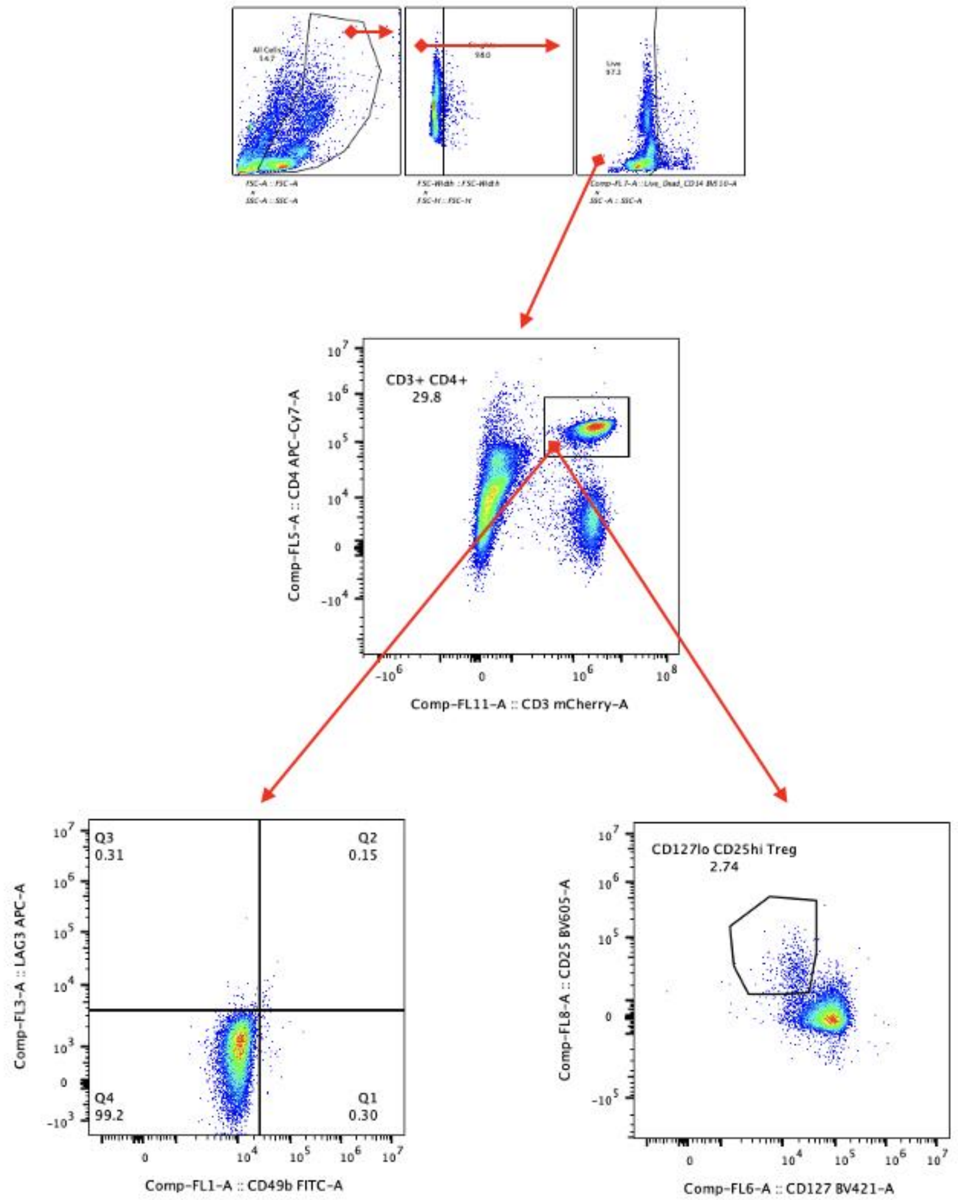

Supplemental Figure 1. Gating strategy for (1A) Panel 1, (1B) Panel 2, and (1C) Panel 3.

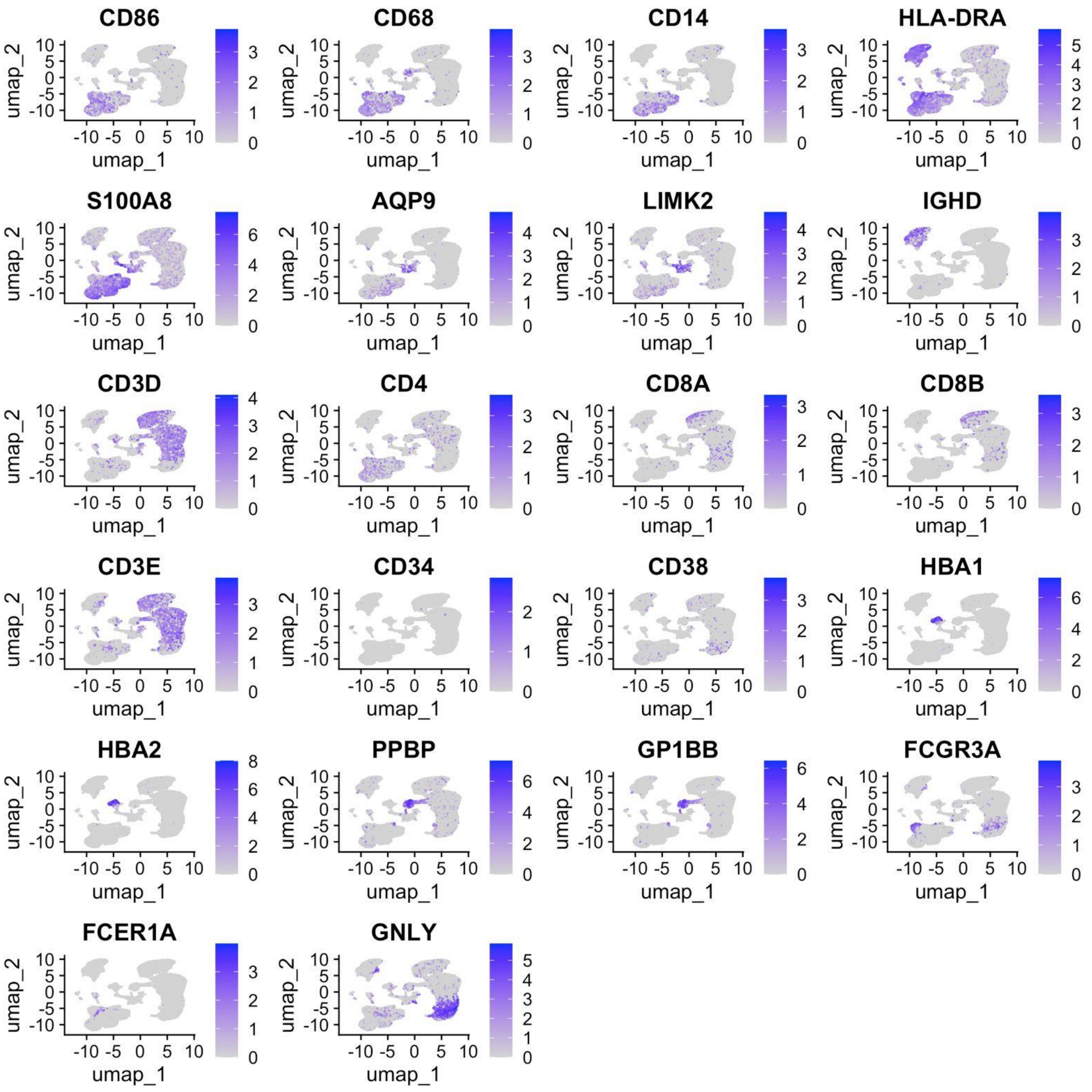

Supplemental Figure 2. UMAPs of the combined PB and UCB dataset depicting gene expression profiles of key lineage-specifying genes. PB: n=3 donors, UCB: n=2 donors.

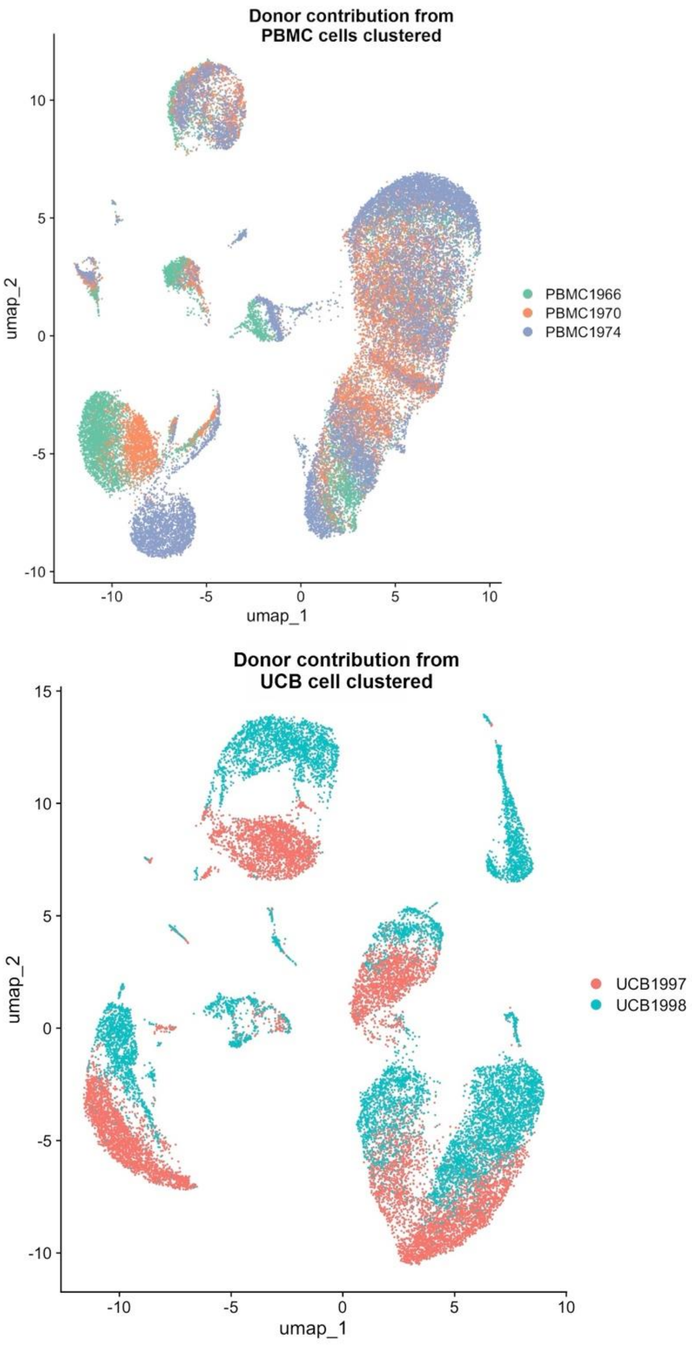

Supplemental Figure 3. UMAPs of clustered UCB (left) and PB (right) cells, coloured by donor. PB: n=3 donors, UCB: n=2 donors.
